# The Place of Local Anæsthesia in Surgery

**Published:** 1898-03

**Authors:** F. B. Lund

**Affiliations:** Boston, Mass.


### THE PLACE OF LOCAL ANÆSTHESIA IN SURGERY.[1]

BY F. B. LUND, M.D., BOSTON, MASS.[2]

Of the advantages offered by local anæsthesia, as compared with general anæsthesia in a large class of surgical operations, there is no need for me to speak before this assembly. Suffice it to say that early in my experience as a medical student I became so convinced of the value of local anæsthesia by hydrochlorate of cocaine, in enabling me to do away with the nauseating effects of general anæsthesia,—the vomiting, dizziness, delirium, and general weakness,—which are so unpleasantly associated with ether in the minds of many patients, that in case of subsequent operations they sometimes prefer, if the operation is a slight one, to suffer it without anæsthesia rather than be etherized, that I have always been an earnest student of methods of local anæsthesia as applied to surgical practice.

---

[1] Remarks before the American Academy of Dental Science, December 1, 1897.

[2] Assistant Visiting Surgeon, Boston City Hospital.

The experiences of eight years in the use of local anæsthesia in minor surgery, for which I have had during the past three years the extensive opportunity furnished by the large out-patient clinic at the Boston City Hospital, have given me such definite views as to the use and abuse of local anæsthesia, the class of cases in which it should be employed, and the technique which is essential to give good results, that I have thought it might not be uninteresting to you, even though I am compelled to confess my entire ignorance of local anæsthesia in dental surgery, to hear what I have found to hold true with regard to its use in a general surgical practice. I have had a certain amount of experience in the use of local anæsthesia in major as well as minor operations, and I think you will agree with me that even in the former it has a distinct and legitimate field.

The various methods of producing local anæsthesia which have been up to the present time employed will be found to divide into but two fundamental classes,—first, those which depend upon numbing the part by freezing mixtures and evaporating spray; and, second, those which depend upon bringing some drug having local anæsthetic properties in contact with the terminal filaments of the sensory nerves. Of local anæsthesia by cold it is not necessary to speak, save to say that, although much older than other methods, it has always failed to find an extensive field of usefulness,—first, owing to the transitory nature of its effects, which renders it unavailable for operations requiring more than a few seconds for their performance; and, secondly, owing to the fact that the processes of freezing and thawing, more particularly the latter, are both exceedingly painful, so that, even if we have anæsthesia or absence of pain during an operation, we certainly do not have it either before or after it. Since 1884, when Köller, of New York, then a medical student in Vienna, discovered the local anæsthetic effects of cocaine upon the mucous membranes, that drug and other synthetical compounds which have recently followed it have vastly extended the field for local anæsthesia in surgery.

It was found that, although cocaine was effective upon mucous membranes by direct absorption from their surface, its use upon intact skin required hypodermic injection in order to be effective. The hypodermic injections in the early days were made with strong solutions,—eight to ten per cent.,—such as were used as applications to mucous membranes; and it was soon found that, owing to the toxic properties of the drug, but small amounts could be safely injected, so that only very brief operations could be performed under cocaine, without danger of producing constitutional effects. Occasional reports of cases of poisoning made surgeons chary of the use of cocaine, and when they did use it, so small was the amount of fluid injected that the operative field was seldom fully permeated with the drug, and anæsthesia often failed to appear. The technique employed was to inject a few drops of the strong cocaine solution in the neighborhood of the field of operation, and then wait for the anæsthetic fluid to soak into the surrounding parts. This procedure was modified by Dr. Leonard J. Corning, of New York, who was the first to demonstrate that solutions of cocaine injected into and around the trunk of a nerve produced anæsthesia in the distribution of its terminal filaments.

At this stage of our knowledge of anæsthesia by cocaine, failures were frequent, for the reason that it was impossible to tell just how far the anæsthetic effect of cocaine had spread, and incisions were frequently carried into non-anæsthetized tissue, greatly to the discomfiture of both patient and surgeon. The latter, as a result of a few experiences of this sort, was often led to give up local anæsthesia and pronounce it a failure; and so great was the prejudice against it that many surgeons even now hold that cocaine and other local anæsthetics give at best uncertain results, that operations under them are never perfectly painless, and general anæsthesia is to be preferred in almost all cases.

But those who were not discouraged by their early failures in local anæsthesia went on perfecting the technique,—Landerer in Germany, Reclus in France, and Halsted in this country being notable examples. It was discovered that weaker solutions of cocaine gave as good results as the stronger, if properly injected, and that the employment of one- and two-per-cent. solutions allowed the performance of more extensive operations without the danger of poisoning. It was observed, as the solutions were injected in larger amounts, that the tissue was saturated, distended, rendered œdematous by the fluid, absolute insensibility to pain was produced, and cutting, scraping, or other manipulations could be performed without any untoward sensation on the patient's part.

Reclus was one of the first to show that by injecting a syringeful of a one-per-cent. solution into the skin, and then beginning the next injection within the area of anæsthesia produced by the first one, quite large operative fields could be rendered anæsthetic.

Recently, Schleich, of Berlin, has elaborated the technique of local anæsthesia, and shown that the injection of a solution of salt and other drugs has an anæsthetic effect, which is dependent upon the distention or infiltration of the tissues with the solution. He is unable, however, in practical work to dispense with cocaine in his anæsthetic solution, for the reason that, although the injection of a simple salt solution produces a local anæsthetic area, the process of injection is in itself painful, unless a little cocaine—1 part in 500 or 1 part in 1000—is added to the solution. Two years ago I published the results of my experience with the use of Schleich's solutions of salt with 1 part in 500 or 1 in 1000 of cocaine and a little morphine, and stated that I had found them perfectly satisfactory in the performance of numerous minor operations, and a few major operations in which, for some reason, the employment of a general anæsthetic was contraindicated. Since that time, acting on the theory that if it was necessary to add cocaine to the salt and morphine solutions in order to prevent pain during their injection, the cocaine alone, being a more definite local anæsthetic than the salt and morphine, ought to be sufficient, I have been employing solutions of cocaine in the strength of 1 in 500 in inflamed tissues, and 1 in 1000 in extensive operations, on non-inflamed tissues, and have found the results to be just as satisfactory as when I employed the more complicated solutions of Schleich. I have, therefore, employed the 1 to 500 and 1 to 1000 solutions of cocaine as a local anæsthetic fluid for routine use in operating under local anæsthesia. Also, during the last twelve months, since the new synthetic compound eucaine has been exploited and advocated as more effective and less toxic than cocaine, I have taken opportunity to try it in a large number of minor operations, and have endeavored to carefully compare it with cocaine. I have found that in the strength of from 1 to 200 to 1 to 500 it had marked local anæsthetic properties, and that it produced anæsthesia which was as complete and about as long continued as that of my cocaine solutions. I have found, however, that in inflamed tissues eucaine solutions are more apt to cause pain during the injection than is the case with cocaine solutions, and that in operations in vascular regions, such as the face, the fact that it causes dilatation of the blood-vessels is a decided disadvantage. As is well known, infiltration with cocaine solutions causes constriction of the smaller blood-vessels, so that during a small operation on the face the surgeon is often not at all hampered by capillary oozing, at least during the first part of the dissection, when he is making his incision or getting down to the capsule of a tumor. When eucaine is employed, the furious bleeding which ensues seriously hampers the surgeon.

I have employed a eucaine solution in the removal of a fatty tumor the size of the fist with perfect success in every respect, except that the hemorrhage was, I think, somewhat more violent than it would have been if cocaine had been employed. For injection into the bladder for the purpose of using the cystoscope, I think I should prefer eucaine, as large amounts of a strong cocaine solution, which, under these conditions are required, are apt to be absorbed by the mucous membranes, and cocaine-poisoning might result. I have never had any toxic effects from the use of cocaine in the weak solutions in which I have employed it for infiltration. I see no reason for discarding it in favor of eucaine, and have, therefore, continued to employ it as a routine anæsthetic. Eucaine is somewhat less expensive than cocaine, and it is claimed that solutions of it can be boiled without decomposing, which is not true of cocaine.

Whether cocaine or eucaine were employed, I have found that my success in securing anæsthesia depended entirely on the careful carrying out of a certain technique. Careless technique always results in pain to the patient and reproach to the surgeon. There are certain operations, and some even of a minor character, in which considerable practice has not enabled me to attain sufficient skill in the technique to avoid causing pain, and in these, therefore, unless contraindicated for some special reason, I should advise the employment of a general anæsthetic. The one essential point in local anæsthesia by cocaine or any other solution, and this was well emphasized by Schleich, is the complete infiltration of the tissues with the anæsthetic fluid. A condition of artificial œdema must be produced, so that the sensitive nerve-filaments are thoroughly surrounded by and saturated with the fluid. In proportion as it is possible to produce this condition practically, will we be successful in our results. The fluid must precede the needle, and the needle the knife, and if we never cut or push our needle or other instrument beyond the territory which has been saturated with the anæsthetic fluid, we can operate without pain to our patient. The method is as follows: the skin being, if loose, pinched up between the thumb and forefinger of the left hand, or, if tense, depressed by pressing down obliquely upon it with the needle-point, the point of the needle is slowly introduced, with the opening, which is cut obliquely, it will be remembered, downward until it is partly through the outer layer of the skin. Then a few drops of the solution is forced in, the posterior part of the opening being closed by pressing the needle downward upon the skin. A few drops having been injected, the needle-point is entirely introduced into, but not through, the skin, and a syringeful of fluid injected, making a raised white wheal, looking like a mosquito-bite. Over this wheal it will be found that the skin is perfectly anæsthetic. Then the needle is withdrawn and introduced within, but near the edge of, the anæsthetic area, and more fluid injected, extending the anæsthetic area in the required direction until sufficient skin area has been covered. If it is desired to remove a wen or small subcutaneous tumor, a circle of these wheals is made around it, and then the needle is pushed under the tumor and the tissues beneath it infiltrated. This process, if properly performed, has caused the patient no pain, or, at most, only a slight pain at the first introduction of the needle. Then the tumor may be dissected out, the knife cuts never extending beyond the area rendered œdematous, without the slightest pain to the patient. In deeper operations, requiring the division of muscle, fascia, and periosteum, after the infiltration of the skin and subcutaneous tissue, these may be cut through, and the fascia and periosteum infiltrated under the guidance of the eye, and then operated upon strictly within the anæsthetized area. In certain conditions this technique is easy, and in others difficult or impossible to carry out, and there are, therefore, certain cases in which we can operate without ether or chloroform and without pain, and others in which we cannot.

Experience with a large number of cases enables one to properly choose those adapted to local anæsthesia, and save his patients the discomfort and danger of general anæsthesia in many operations of minor surgery. It also enables him to avoid the discomfiture which results from promising to operate without pain in cases in which he is unable to carry out his project, and either has to hurt his patient badly or, after the delay caused by an unsuccessful attempt, to have recourse to general anæsthesia.

Any transgression beyond the anæsthetized area will cause pain to the patient, and in operating in inflamed tissues the pulling upon the inflamed area by retractors and the like, even if they be in contact only with anæsthetized tissues, will cause pain and difficulty.

In general, the deeper and more complicated the dissection, the more difficult and irksome will be the carrying out of a satisfactory technique. In some cases, where large nerve-trunks have to be cut across, I have found the infiltration difficult to carry out.

Schleich recommends the anæsthetization of nerves by the application of a drop of strong carbolic acid to their divided ends, but such a procedure certainly does not mitigate the pain of their division, which is severe, and I have found sometimes that I came upon non-anæsthetized nerves in areas which were otherwise satisfactorily infiltrated, and that their section caused so much pain that I wished that I had operated under general anæsthesia. The reason for the failure of infiltration to reach the larger nerve-trunks, I think, is undoubtedly the fact that the filaments contained in them are so protected by their sheaths that the fluid does not reach them unless the needle has been actually introduced into the sheath, a procedure which we cannot perform with accuracy unless the nerve is freely exposed in the dissection, and which even then is apt to be attended with pain. Operations upon the median line of the body are especially adapted to deep dissections under local anæsthesia, for the reason that no large nerve-fibres are met with, and we have to do only with the terminal filaments which meet in the middle line.

This applies to such operations as median laparotomy, perineorrhaphy, and the like. Such operations, as will be explained more in detail below, I would not advocate performing in general under local anæsthesia; but in special cases and emergencies, when a general anæsthetic is inadvisable, they may be performed with considerable facility and freedom from pain under local anæsthesia.

A brief classification of operations, according to the facility with which they may be performed under local anæsthesia, with remarks explanatory of the reasons, will perhaps enable us to appreciate the limits of the applicability of local anæsthesia better than any other method. In Class 1 I would place those operations in which local anæsthesia gives in general perfectly satisfactory results, and in which I would rarely employ a general anæsthetic except for special reasons, such as idiosyncrasy of the patient or the desire on the part of the patient not to be conscious that he is being operated upon. Such operations are the removal of subcutaneous tumors in non-inflamed tissue, particularly in the scalp, face, back of the neck, or median line of the body. Epitheliomata, either upon the face or anywhere upon the body, may be easily and satisfactorily removed under local anæsthesia. Large tumors of this character may often be satisfactorily removed. The skin of the back, particularly near the median line, is very easy to render anæsthetic, as it is sparsely provided with sensitive nerve-filaments. Small circumscribed abscesses, especially those formed by the breaking down of tubercular glands, which are so thoroughly softened as not to require extensive curetting, may be opened and drained under cocaine absolutely without pain.

The removal of foreign bodies from the extremities, such as needles in the hand, and operations for ingrowing toe-nail, can be performed under cocaine with perfect ease, and for these I rarely employ general anæsthesia. Felons of the fingers may be done under cocaine if the inflammation has not extended beyond the second joint, so that the infiltration can be begun in healthy tissue. All the tissues of the fingers and toes may be infiltrated, including skin, subcutaneous tissue, tendons, and periosteum, so that the bone may be amputated without pain. Where bone operations are to be performed, it is frequently wiser to employ a general anæsthetic, as the crunching of bone-forceps and sawing of bone is disagreeable to many patients, even though unattended with pain.

In the removal of pieces of tumors for diagnoses, local anæsthesia has an important place. In general, with regard to operating upon the skin and subcutaneous tissues, it will be found that where the skin and underlying tissues are soft and not stiffened by callous or tough fascia, infiltration is easy. This is true of the skin over nearly the whole of the body.

In the palms of the hands and soles of the feet, the toughness of the skin and its adhesion to the strong underlying fascia render infiltration difficult, owing to the force required to send the fluid into the tissues, and also the impossibility of judging just how far it has gone.

In Class 2 I would place a class of cases in which local anæsthesia, although not giving ideal results, is often of advantage. Here comes moderately extensive abscesses, where curetting is not required. Such abscesses may be infiltrated and incised absolutely without pain; but the dilatation of the wound and insertion of the drainage-tube are painful, owing to the stretching of the inflamed tissues beyond the anæsthetized area.

In Class 3, operations in which local anæsthesia should not in general be employed, I should place operations upon extensive inflammatory processes, which it is impossible to infiltrate and operate upon without pain, as the tissues are already so distended with the serum as the result of inflammation that further infiltration is impossible. The cocaine cannot be driven in without first driving out the serum from the already distended lymph-space, a process which will often be attended with as much pain as operations without anæsthesia.

In general, the only operations which can be performed with perfect local anæsthesia on inflamed tissues are those in which it is possible to surround the inflamed area by an infiltration in healthy tissues cutting off its nerve-supply. Extensive dissections of the inguinal glands for adenitis have been reported by Bransford Lewis and other authors, as performed under local anæsthesia; but in my own experience, no matter how careful the technique, I have found local anæsthesia unsatisfactory in these cases. In this class of operations, which should not be attempted under local anæsthesia,—that is, in the majority of instances,—should be placed major operations in general, and those which require consultation during the operation between the operator and his assistants or consultants, a participation in which might be demoralizing to the patient. Operations involving exposure and disagreeable manipulations about the genitals or rectum are usually, in sensitive persons, best performed under general anæsthesia. In deep dissections around important blood-vessels and nerves it is usually advisable to employ a general anæsthesia.

As to abdominal operations, the necessity for muscular relaxation, and the extent and formidable nature of the manipulation required, render them unsuitable for local anæsthesia. In operations for rupture of adhesions about ankylosed joints and other procedures requiring stretching and straining of adhesions, local anæsthesia is of no benefit. In delicate plastic operations, such as those about the eyelids, the infiltration anæsthesia so distorts the flaps as to increase the difficulties of the operation, and therefore it is a rule better to resort to a general anæsthetic.

In the above list of operations in which local anæsthesia is not in general advised are included so large a number of the major operations of surgery, that we may well inquire whether local anæsthesia has any field among operations of a more formidable character. The answer is, that in certain cases of a major character, some of these among the more important surgical emergencies, local anæsthesia has proved of the greatest value.

These are operations which we often have to perform under a vital indication, and in which the employment of a general anæsthetic adds greatly to the danger of losing the patient. In these cases we can often save our patient a vast amount of suffering by the use of local anæsthesia.

The operation of tracheotomy, where required for stenosis of the larynx, may be easily and painlessly performed under cocaine, and the obvious dangers of a general anæsthetic avoided. The dangers of general anæsthesia in operations for neglected strangulated hernia in patients the subjects of chronic bronchitis are well known. The operation for the relief of strangulated hernia may be easily and satisfactorily performed under local anæsthesia. I was recently called upon to operate upon a recently strangulated femoral hernia as large as a fœtal head at term in a woman of sixty years with a weak heart, beating at the rate of 160, who also had a chronic bronchitis. Under local anæsthesia I was able with ease not only to open the sac, incise the constricting ring, reduce the two and a half feet of distended intestine, with the cæcum and appendix, which were contained in the hernia, but to suture the ring, so that I hope a radical cure has been effected. The operation was performed two weeks ago, and the woman's recovery has been uneventful. I felt that general anæsthesia would have added very greatly to the danger of this operation, which was performed with so little pain to the patient that she thanked me at the close of the proceeding for hurting her so little. Intestines can be handled or even incised without pain even without an anæsthetic. I have anæsthetized the abdomen of an old man, the state of whose kidneys would not allow of general anæsthesia, for Dr. H. W. Cushing, who performed a suprapubic cystotomy, and have seen Dr. Gerster perform a gastrotomy for cancer of the œsophagus under cocaine in a patient too weak for general anæsthesia, and immediately feed the patient through the opening before he left the operating-table.

The chest may be drained under local anæsthesia in empyema, in cases where the pressure of the fluid on the heart made general anæsthesia inadvisable.

In view of these facts, although local anæsthesia is not in general advisable for major operations, there is a certain class in which it is not only advisable, but demanded, and its employment in preference to a general anæsthetic may save the patient's life.

One word with regard to the employment of local anæsthesia in neurasthenic patients. There is a certain class of patients, mostly neurotic women, who desire not only not to feel the pain of the knife, but have such a horror of surgery that the mere knowledge that they are being cut, even though the cutting be painless, will cause them to struggle, protest, and make outcry. After making a tremendous outcry and greatly disconcerting the surgeon, these patients will often tell him that they felt no pain, but the consciousness that they were being operated upon, and the fear that they would be hurt, were so dreadful that they wished they had taken ether. In patients of this sort it is wise to employ a general anæsthetic.

